# Plasma Calmodulin as a Biomarker of Subclinical Cardiovascular Disease in Pediatric Chronic Kidney Disease

**DOI:** 10.3390/children12050599

**Published:** 2025-05-04

**Authors:** Hsin-Jung Lee, Wei-Ting Liao, Chien-Ning Hsu, You-Lin Tain, Pei-Chen Lu

**Affiliations:** 1 Division of Pediatric Nephrology, Kaohsiung Chang Gung Memorial Hospital, Kaohsiung 833, Taiwan; h2222@cgmh.org.tw (H.-J.L.); winona0409@cgmh.org.tw (W.-T.L.); tainyl@cgmh.org.tw (Y.-L.T.); 2 Department of Pharmacy, Kaohsiung Chang Gung Memorial Hospital, Kaohsiung 833, Taiwan; cnhsu@cgmh.org.tw; 3 School of Pharmacy, Kaohsiung Medical University, Kaohsiung 807, Taiwan; 4 College of Medicine, Chang Gung University, Taoyuan 333, Taiwan; 5 Department of Pediatrics, Kaohsiung Municipal Ta-Tung Hospital, Kaohsiung 801, Taiwan

**Keywords:** children, chronic kidney disease, hypertension, cardiovascular disease, calmodulin, biomarker

## Abstract

Background: Calmodulin is a calcium-signaling protein implicated in cardiac remodeling and could be released extracellularly. It was previously identified as differentially expressed in hypertensive pediatric chronic kidney disease (CKD). This study assessed plasma calmodulin as a cardiovascular disease (CVD) biomarker in pediatric CKD and compared it with traditional risk markers. Methods: We conducted a cross-sectional study of 81 children with CKD aged 3–18 years. All underwent clinical assessments and echocardiography; 44 had carotid ultrasound, and 38 completed ambulatory blood pressure monitoring (ABPM). Results: Most participants had preserved renal function (median eGFR, 104.4 mL/min/1.73 m^2^). Plasma calmodulin levels were significantly associated with early markers of CVD, including interventricular septal thickness, left ventricular mass, carotid intima–media thickness, and ABPM systolic measures (all r > 0.2; *p* < 0.05). In multivariable analysis, only calmodulin and office systolic blood pressure (BP) independently predicted abnormal BP profiles. Conclusions: Plasma calmodulin may serve as a sensitive, though non-specific, early CVD biomarker in pediatric CKD and could complement conventional screening tools.

## 1. Introduction

Cardiovascular disease (CVD) is the leading cause of morbidity and mortality in patients with chronic kidney disease (CKD), and this burden begins early in life. In pediatric CKD, cardiovascular complications often present subclinically, such as elevated arterial stiffness [1,2], left ventricular hypertrophy [2,3,4], or abnormal 24 h ambulatory blood pressure monitoring (ABPM) patterns [5], long before overt events occur. Early identification of cardiovascular risk in this population is crucial, but conventional clinical tools remain limited in sensitivity and specificity.

Numerous circulating biomarkers reflecting diverse pathophysiological mechanisms—such as endothelial dysfunction, kidney injury, inflammation, and oxidative stress—have been proposed as potential indicators of CVD in children with CKD [6]. Markers like ADMA [7,8], FGF23 [9,10,11], ApoC-II [12], and indoxyl sulfate [13] have shown promise, yet few have translated into clinically actionable tools due to overlapping expression across diseases or inconsistent predictive power in children.

In our previous iTRAQ-based plasma proteomic study [12], we identified calmodulin, a calcium-binding intracellular signaling protein, as differentially expressed in pediatric CKD with hypertension. Calmodulin plays a central role in regulating vascular tone, myocardial contraction, and ion channel function [14,15]. While it is well-established as an intracellular messenger with high transcriptional expressions in tissue, such as brain and cardiomyocytes, its presence in plasma is atypical and may reflect cellular stress or injury—particularly under pathological conditions such as CKD or CVD. Notably, no studies to date have evaluated plasma calmodulin as a biomarker in pediatric CKD or examined its relevance to subclinical cardiovascular remodeling.

Although hypertension is a recognized clinical marker of CVD in pediatric CKD and is routinely monitored according to Kidney Disease: Improving Global Outcomes (KDIGO) 2024 guidelines [16], traditional blood pressure (BP) assessments (e.g., office BP) may not adequately capture early or subclinical cardiovascular changes [17]. While 24 h ABPM offers greater sensitivity [18], it is currently recommended only for children with CKD stage 3 or above [16]. Moreover, these guidelines do not advocate routine use of imaging modalities such as echocardiography or carotid artery ultrasound [16]. Therefore, the identification of complementary biomarkers that reflect early cardiovascular abnormalities may improve the accuracy of risk stratification in this high-risk population.

We hypothesize that plasma calmodulin may serve as a sensitive, though not disease-specific, indicator of early CVD in children with CKD. This study aims to investigate the association between plasma calmodulin levels and cardiovascular phenotypes—including ABPM parameters, arterial stiffness indices, and echocardiographic measurements in pediatric CKD. By doing so, we hope to determine the potential role of circulating calmodulin in CVD risk stratification in this high-risk group.

## 2. Materials and Methods

### 2.1. Study Design and Participants

This cross-sectional study was conducted in accordance with the ethical principles outlined in the 1964 Declaration of Helsinki and its subsequent amendments, and was approved by the Institutional Review Board (IRB) of Chang Gung Medical Foundation, Taoyuan, Taiwan (approval numbers: 201701735A3C501, 202001973A3C601, and 202001973A3C503). Written informed consent was obtained from all participants and their legal guardians prior to study participation. Children and adolescents with chronic kidney disease (CKD), aged 3 to 18 years, were recruited from the outpatient clinics at Kaohsiung Chang Gung Memorial Hospital between August 2021 and December 2024. CKD diagnosis and staging were performed according to the Kidney Disease Outcomes Quality Initiative (K/DOQI) guidelines issued by the National Kidney Foundation [19]. Renal function was assessed using the bedside CKiD equation, which estimates the glomerular filtration rate (eGFR) based on the patient’s height and serum creatinine concentration [20].

The exclusion criteria were (a) eGFR < 15 mL/min/1.73 m^2^; (b) current dialysis treatment; (c) history of kidney transplantation; (d) pregnancy; (e) congenital heart disease; (f) failure to complete follow-up; and (g) inability to adhere to study protocols. The final analysis included participants with baseline eGFR > 15 mL/min/1.73 m^2^. Detailed renal and cardiovascular assessments were conducted as described below.

### 2.2. Clinical Assessments and Specimen Collection

During a single clinic visit, each participant received a comprehensive assessment, including (a) review of medical history, measurement of office BP, and physical examination; (b) laboratory evaluations of blood and urine samples; (c) transthoracic echocardiography; (d) 24 h ABPM; and (e) carotid ultrasonography. The causes of CKD were classified as either congenital anomalies of the kidney and urinary tract (CAKUT) or non-CAKUT origins. Specific CAKUT diagnoses encompassed conditions such as renal hypoplasia, renal dysplasia, unilateral renal agenesis, reflux nephropathy, polycystic kidney disease, horseshoe kidney, duplex kidney, posterior urethral valves, and various ureteral abnormalities. Non-CAKUT was defined as all other causes of kidney disease, including isolated hematuria or proteinuria. Proteinuria was defined as a urine protein-to-creatinine ratio (UPCR) ≥ 200 mg/g. Hematuria was defined as ≥5 red blood cells per high-power field in a centrifuged urine specimen on at least two separate occasions.

### 2.3. Anthropometric Measurements

Body mass index (BMI), calculated as weight in kilograms divided by height in meters squared (kg/m^2^), was used to assess weight status in children and adolescents. Given the ongoing growth and development during childhood and adolescence, BMI was interpreted using age- and sex-specific percentiles based on the reference standards published by the Taiwan Ministry of Health and Welfare [21]. According to these national growth charts, underweight was defined as a BMI below the 5th percentile, overweight as a BMI greater than the 85th percentile, and obesity as a BMI greater than the 95th percentile, based on age and sex.

### 2.4. Office BP and ABPM

After resting for five minutes, office BP measurements were taken three times using a validated electronic device, and the mean value of the three readings was utilized for further analysis. BP evaluation adhered to the 2017 guidelines from the American Academy of Pediatrics [22]. Elevated BP was characterized by systolic or diastolic values between the 90th and 95th percentiles, or by 120/80 mmHg if lower, for children aged 1 to <13 years, and by systolic 120–129 mmHg with diastolic pressure < 80 mmHg for adolescents aged ≥13 years. Hypertension was defined as systolic or diastolic BP ≥ 95th percentile for children aged 1 to <13 years, and as BP ≥ 130/80 mmHg for those aged ≥13 years.

Participants aged 6–18 years underwent 24 h ABPM within one week of their clinic appointment, utilizing an Oscar 2 ABP device (SunTech Medical Inc., Morrisville, NC, USA). Both BP and heart rate were measured every 20 min throughout a 24 h cycle during participants’ normal activities. Abnormal ABPM parameters were defined based on five criteria: (1) 24 h hypertension, indicated by a mean 24 h systolic or diastolic BP exceeding the 95th percentile or ≥130/80 mmHg; (2) daytime (awake) hypertension, defined as a mean daytime BP above the 95th percentile; (3) nocturnal (asleep) hypertension, defined as a mean nighttime BP above the 95th percentile; (4) elevated BP load, defined as more than 25% of systolic or diastolic BP readings exceeding the 95th percentile; and (5) a non-dipping pattern, characterized by a <10% decline in mean nocturnal BP compared to daytime values. Participants who met any one or more of these criteria were classified as having an abnormal ABPM pattern.

### 2.5. Echocardiography and Carotid Artery Ultrasonography

Both echocardiography and carotid artery ultrasonography were conducted on the same day as office BP assessments. Pediatric cardiologists (I-Chun Lin and Mao-Hung Lo) performed the echocardiographic examinations, whereas pediatric nephrologists (Wei-Ting Liao and Wei-Ling Chen) carried out the carotid scans using a 5–12 MHz linear array probe with the ProSound α7 ultrasound device and eTRACKING software (Aloka Co., Tokyo, Japan).

### 2.6. Plasma Calmodulin Measurement

Plasma calmodulin concentrations were measured using a commercially available sandwich ELISA kit (Human Calmodulin ELISA Kit, Novus Biologicals, Centennial, CO, USA; NBP2-74974) in accordance with the manufacturer’s protocol. Blood samples were collected into EDTA tubes, processed by centrifugation at 1000× *g* for 15 min at 2–8 °C within 30 min after collection. The assay utilized a double-antibody sandwich approach, with ELISA plates pre-coated with a monoclonal antibody targeting human calmodulin. Following incubation, biotinylated detection antibodies, and horseradish peroxidase-labeled avidin were sequentially added. Absorbance at 450 ± 2 nm was read by a microplate reader. All standards and samples were tested in duplicate, and concentrations were determined by comparing absorbance to a standard curve created through serial dilutions of a known calmodulin standard.

### 2.7. Statistical Analysis

Statistical analyses were conducted using GraphPad Prism version 10.4.1 (GraphPad Software, LLC, San Diego, CA, USA). Continuous data were expressed as medians with IQR, while categorical variables were summarized as frequencies and percentages. The Mann–Whitney U test was applied to assess differences between groups. Pearson correlation was performed to evaluate the relationships between plasma calmodulin levels and clinical parameters. Logistic regression analysis was conducted to identify predictors of abnormal BP profiles. Variables achieving a *p* value < 0.1 in univariate analysis were entered into multivariate models, and multicollinearity was assessed by calculating variance inflation factors (VIFs). Predictive performance was evaluated using receiver-operating characteristic (ROC) curve analysis, and the Youden index was applied to determine the optimal cutoff value for plasma calmodulin.

## 3. Results

### 3.1. Participant Enrollment and Data Collection

**Figure 1:**
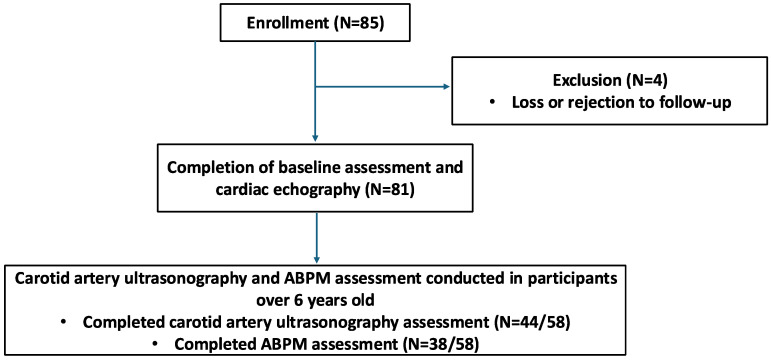
Flowchart of participant enrollment and assessment procedures.

A total of 85 children aged 3–18 years with CKD were enrolled in this study (Figure 1). Four participants were excluded due to loss to follow-up or refusal to participate, resulting in a dropout rate of 4.7%. The final cohort included 81 participants who completed baseline assessments and echocardiography. Among those older than 6 years (n = 58), additional evaluations were conducted, including carotid artery ultrasonography and 24 h ABPM. Of these, 44 participants completed carotid artery ultrasonography assessments, and 38 completed ABPM.

### 3.2. Participant Characteristics

Of the 81 participants (Table 1), 47 (58.0%) were male, and the median age was 9.48 years [IQR: 6.70–12.86]. The primary diagnosis was CAKUT (n = 60, 74.1%), including unilateral renal agenesis (n = 31), hydronephrosis (n = 8), renal cyst (n = 8), unilateral renal dysplasia (n = 8), and vesicoureteral reflux with reflux nephropathy (n = 5). The remaining participants were diagnosed with non-CAKUT conditions, including nephrotic syndrome (n = 8), dense deposit disease (n = 1), isolated proteinuria (n = 4; UPCR 200–2000 mg/g), and isolated hematuria (n = 8). Further details of UPCR values are provided in Table S1. Overall, kidney function was preserved, with a median eGFR of 104.4 mL/min/1.73 m^2^ [IQR: 90.31–117.2].

#### 3.2.1. Traditional Cardiovascular Risk Factors

Traditional cardiovascular risk factors were highly prevalent in this cohort (Table 1). Overweight or obesity was observed in 32.1% (n = 26), and elevated office BP or hypertension was noted in 61.7% (n = 50). Regarding lipid profiles, the median low-density lipoprotein cholesterol (LDL-C) was 93.0 mg/dL [IQR: 75.25–118.5], with levels above 100 mg/dL considered abnormal. The median triglyceride level was 67.0 mg/dL [IQR: 45.0–97.25], with values >90 mg/dL classified as abnormal.

Other metabolic parameters were generally within the normal range. Median fasting plasma glucose was 85.5 mg/dL [IQR: 82.0–89.75] (normal range: 70–99 mg/dL), and the median uric acid level was 4.8 mg/dL [IQR: 4.2–6.1] (normal range: <7 mg/dL).

#### 3.2.2. CKD-Specific Cardiovascular Risk Factors

Most CKD-specific cardiovascular risk factors were within normal ranges (Table 1). The median UPCR was 54.9 mg/g [IQR: 38.75–93.35] (reference: <200 mg/g). Median serum calcium and phosphate levels were 10.1 mg/dL [IQR: 9.8–10.4] and 4.8 mg/dL [IQR: 4.4–5.1], respectively. The median hemoglobin was 13.1 g/dL [IQR: 12.65–14.3], and the median white blood cell (WBC) count was 6.6 × 10^3^/μL [IQR: 5.65–8.1], indicating no overt anemia or systemic inflammation.

#### 3.2.3. Plasma Calmodulin Levels

Calmodulin, a calcium-binding messenger protein potentially involved in endothelial function and vascular regulation, showed a median plasma level of 137.9 × 10^2^ pg/mL [IQR: 83.35–201] (Table 1). Its role in CKD-related cardiovascular dysfunction warrants further investigation.

### 3.3. Correlation Between Calmodulin and Baseline Characteristics

To better understand the physiological context of plasma calmodulin and minimize potential confounding bias, we conducted subgroup comparisons and correlation analyses. Mann–Whitney U tests (Table S2) showed no significant differences in calmodulin levels based on gender or kidney disease cause (CAKUT vs. non-CAKUT), suggesting that neither gender nor underlying renal pathology was associated with plasma calmodulin levels.

Pearson correlation analysis (Table 2) revealed that plasma calmodulin levels were significantly positively correlated with BMI (r = 0.3265, *p* = 0.0031), body weight (r = 0.2717, *p* = 0.0141), and serum calcium (r = 0.3268, *p* = 0.0029). No significant associations were found with age, office BP, lipid profile, fasting plasma glucose, uric acid, renal function, hematological parameters, or other electrolytes (all *p* > 0.05).

### 3.4. Correlation Between Calmodulin and Cardiovascular/Imaging Parameters

To investigate the association between calmodulin and early cardiovascular changes, we analyzed its correlation with echocardiographic, carotid artery ultrasonography, and ABPM findings (Table 3).

#### 3.4.1. Echocardiography

Calmodulin was positively correlated with interventricular septal end-diastolic thickness (IVSd) (r = 0.2492, *p* = 0.0258) and left ventricular mass (LV mass) (r = 0.2472, *p* = 0.0271) (Table 3). No significant associations were observed with left ventricular posterior wall thickness at end-diastole (LVPWd), left ventricular internal diameter at end-diastole (LVIDd), ejection fraction (EF), or fractional shortening (FS).

#### 3.4.2. Carotid Artery Ultrasonography

Calmodulin was positively correlated with right carotid intima–media thickness (IMT) (r = 0.3836, *p* = 0.0104) (Table 3), while no significant correlations were observed with left carotid IMT, left and right carotid β-stiffness index (left/right carotid beta), left and right carotid augmentation index (left/right carotid AI), or left and right carotid pulse wave velocity (left/right carotid PWV).

#### 3.4.3. 24 h ABPM

Calmodulin levels were significantly positively correlated with multiple systolic blood pressure (SBP) parameters (Table 3). These included overall SBP (r = 0.4934, *p* = 0.0016), percentage of overall SBP readings above the 95th percentile (overall SBP load) (r = 0.4746, *p* = 0.0026), awake SBP (r = 0.5094, *p* = 0.0011), and percentage of awake SBP readings above the 95th percentile (awake SBP load) (r = 0.4955, *p* = 0.0016). Significant correlations were also observed with asleep SBP (r = 0.3798, *p* = 0.0187) and percentage of asleep SBP readings above the 95th percentile (asleep SBP load) (r = 0.3681, *p* = 0.0230). No significant correlations were found between calmodulin levels and diastolic blood pressure (DBP), percentage of DBP readings above the 95th percentile (DBP load) during overall/awake/sleep, mean BP, percentage drop in SBP/DBP during sleep compared to awake period (SBP/DBP Asleep Dipping percentage), or the ambulatory arterial stiffness index (AASI).

### 3.5. Association Between Clinical Variables and Abnormal BP

Participants were classified into two groups based on their BP status. The abnormal BP profile group included individuals who met any of the criteria for office BP elevation, office hypertension, or abnormal ABPM patterns as defined above, or who were receiving antihypertensive medications. The normal BP profile group consisted of participants with both office BP and ABPM within normal limits and not on antihypertensive treatment.

Univariate logistic regression was performed to examine associations between clinical variables and abnormal BP profile (Table 4). Significant associations were observed for BMI (OR = 1.158, *p* = 0.0339), office SBP (OR = 1.10, *p* = 0.0013), office DBP (OR = 1.078, *p* = 0.0361), and calmodulin (OR = 1.008, *p* = 0.0167). Other variables, including age, male (vs. female), CAKUT (vs. non-CAKUT), eGFR, lipid profile, uric acid, UPCR, electrolytes, and hemoglobin, were not significantly associated with abnormal BP.

A total of five variables with *p* < 0.1 in univariate analysis (BMI, office SBP, office DBP, uric acid, and calmodulin) were initially considered for multivariable analysis (Table 4). Variance inflation factor testing showed no multicollinearity (all VIFs < 5; Table S3). Between office SBP and DBP, only office SBP was retained in the final model due to its stronger association with abnormal BP and greater clinical relevance. Thus, four variables (BMI, office SBP, uric acid, and calmodulin) were included in the multivariate logistic regression model.

In the multivariate model (Table 4), office SBP (adjusted OR = 1.14, *p* = 0.001) and calmodulin (adjusted OR = 1.012, *p* = 0.029) remained independently associated with abnormal BP.

### 3.6. Predictive Performance of Calmodulin for Abnormal BP

**Figure 2:**
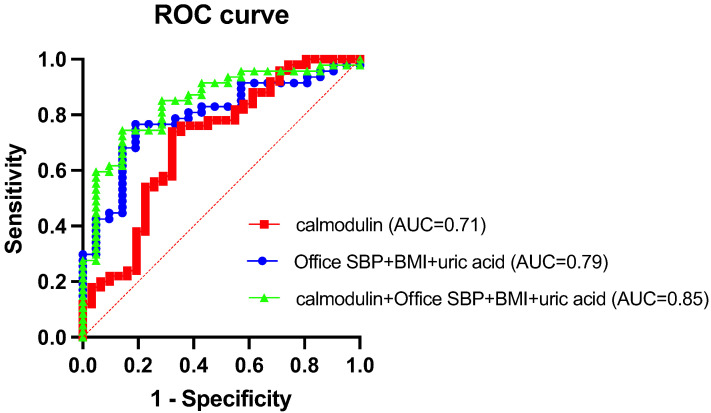
Receiver operating characteristic (ROC) curves evaluating the predictive performance of different models for abnormal BP profiles in children with CKD. The red dashed diagonal line represents a random classifier (AUC = 0.5).

To further assess the predictive utility of calmodulin, an ROC curve analysis was performed (Figure 2). Calmodulin alone yielded an area under the curve (AUC) of 0.71, indicating acceptable discriminatory power. A model combining traditional risk factors (office SBP, BMI, and uric acid) achieved an AUC of 0.79. When calmodulin was added to this model, the AUC improved to 0.85, suggesting that calmodulin provides additional predictive value beyond conventional clinical parameters.

### 3.7. Diagnostic Value of Plasma Calmodulin

Using the ROC curve and the Youden index (Table S4), the optimal cut-off value for calmodulin to identify abnormal BP profile was determined as 131.04 × 10^2^ pg/mL. At this threshold, calmodulin demonstrated a sensitivity of 74%, specificity of 67.74%, positive predictive value (PPV) of 78.72%, and negative predictive value (NPV) of 61.76% (Table 5). These findings suggest a possible association between plasma calmodulin and early cardiovascular changes, though the evidence remains preliminary.

## 4. Discussion

This study investigated the association between plasma calmodulin and cardiovascular risk markers in children with CKD. Our findings revealed that calmodulin levels were significantly correlated with systolic parameters on ABPM, as well as with LV mass, IVSd, and right carotid IMT. Furthermore, calmodulin independently predicted abnormal BP profiles even after adjusting for conventional cardiovascular risk factors. When incorporated into a traditional risk model, calmodulin improved its predictive performance, suggesting its potential utility as a biomarker for early CVD in pediatric CKD.

Calmodulin is encoded by three distinct genes (CALM1, CALM2, and CALM3), all of which produce an identical amino acid sequence—highlighting its evolutionary conservation and essential role in calcium signaling. As a ubiquitously expressed intracellular calcium-binding messenger protein, calmodulin participates in numerous physiological processes and is typically not associated with disease-specific expression patterns. Historically, the detection of circulating calmodulin has been attributed to pre-analytical artifacts, either due to its lack of classical secretion signals (e.g., an N-terminal signal peptide) or to non-specific release from platelets during coagulation [23]. However, recent studies suggest that plasma calmodulin may instead reflect pathological conditions such as cellular injury [24] and exosome-mediated release [25]. For example, a study by Esteras et al. on Alzheimer’s disease (AD) reported significantly elevated calmodulin levels in both plasma and peripheral cells of AD patients, achieving 89% sensitivity and 82% specificity in distinguishing AD from healthy controls [26]. Notably, this elevation was not seen in other neurodegenerative diseases, implying that calmodulin may exhibit a degree of disease specificity beyond being a general inflammatory marker. These findings suggest that conditions involving AD and systemic cellular stress may also elevate plasma calmodulin levels, potentially limiting its specificity for cardiovascular outcomes in CKD. Therefore, elevated calmodulin levels should be interpreted with caution and considered in the broader clinical context, especially when other causes of cellular injury may coexist. In our study, children with CKD and abnormal BP profiles showed higher plasma calmodulin levels than those with normal BP. These levels also correlated with cardiac structural changes and carotid remodeling, supporting calmodulin’s role as a sensitive, albeit non-specific, biomarker of early CVD.

We demonstrated that besides office SBP, plasma calmodulin also retains independent predictive value for abnormal BP profiles in children with CKD, even after adjusting for potential confounders. Calmodulin exhibited good discriminatory performance, with a sensitivity of 74%, specificity of 67.74%, PPV of 78.72%, and NPV of 61.76%. The relatively high sensitivity and PPV suggest that calmodulin can effectively identify most children with true BP abnormalities and provide a reliable positive prediction. However, its moderate specificity and NPV indicate a non-negligible proportion of false positives and false negatives. According to the KDIGO guidelines [16], routine cardiovascular evaluation is recommended beginning at CKD stage 3. However, our findings suggest that plasma calmodulin levels may increase during earlier CKD stages (stages 1–2), indicating potential subclinical cardiovascular changes. This highlights the potential role of calmodulin as an early biomarker to identify patients who may benefit from earlier cardiovascular assessment. Notably, traditional cardiovascular risk markers, such as BMI, lipid profiles, and glucose levels, did not show independent associations with BP abnormalities in our pediatric CKD cohort. This may be due to the strong influence of age, gender, hormonal fluctuations, and developmental stage in children, which may undermine the predictive stability of these conventional markers in cross-sectional analyses.

**Figure 3:**
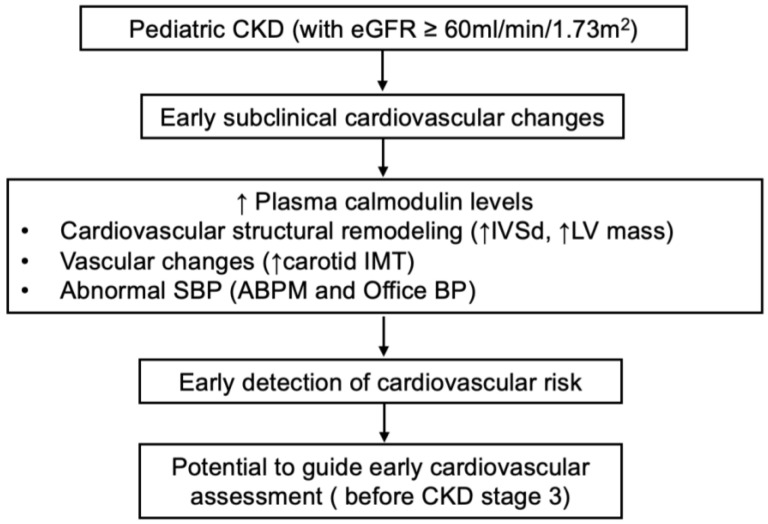
Integrative scheme of study findings.

Although our study revealed significant correlations between plasma calmodulin levels and subclinical cardiovascular parameters, we acknowledge that the current evidence is exploratory and does not establish a causal relationship. Nonetheless, animal and cell studies have demonstrated that calmodulin and its downstream kinases play critical roles in BP regulation and the development of cardiovascular pathology. As a primary intracellular calcium sensor, calmodulin activates multiple downstream effectors, including Ca^2+^/calmodulin-dependent protein kinase II (CaMKII) [27,28], eukaryotic elongation factor 2 kinase (eEF2K) [29], and death-associated protein kinase 3 (DAPK3) [29], which participate in various physiological and pathological processes in vascular smooth muscle cells such as contraction, proliferation, inflammation, and remodeling. In spontaneously hypertensive rats, calmodulin activity has been found to be significantly elevated in the heart and kidney tissue, even in the absence of overt renal dysfunction [30]. This suggests that renal calmodulin activity upregulation may reflect an early response to hemodynamic or oxidative stress rather than structural kidney damage. Given calmodulin’s regulatory role in calcium handling within renal tubular cells—including modulation of TRPV5 channels and calcium reabsorption [31]—its activation in the kidney may be linked to intrarenal calcium signaling disturbances driven by hypertension. Moreover, CaMKII in vascular smooth muscle is activated by vasoconstrictors such as angiotensin II, promoting intracellular calcium elevation and sympathetic excitation, thereby contributing to the development of hypertension [27,28]. In central nervous system regions such as the subfornical organ (SFO), CaMKII has also been identified as a redox-sensitive signaling molecule involved in angiotensin II–induced neuronal activation and BP elevation [32]. Collectively, these studies provide strong evidence supporting the potential pathogenic role of calmodulin and its associated kinases in hypertension. Additionally, the downstream effector, CaMKII is also known to mediate chronic cardiac remodeling [33,34], including hypertrophy, apoptosis, inflammation, and fibrosis [34]. Persistent CaMKII activation has been implicated in the development of arrhythmias and heart failure [35], positioning calmodulin as a core integrator of mechanical and oxidative stress signaling in the myocardium. As previously mentioned, calmodulin may also be released extracellularly via non-classical pathways, including exosome-mediated secretion [25], which further supports its role as a circulating biomarker in stress-related conditions. This may explain why plasma calmodulin positively correlates with subclinical CVD patterns in our study. An integrative scheme summarizing the proposed role of plasma calmodulin in early cardiovascular changes among pediatric CKD patients is presented in Figure 3.

This study has several limitations. First, its cross-sectional design precludes any inference of causality between plasma calmodulin levels and cardiovascular phenotypes. Second, the relatively small sample size—particularly for the ABPM and carotid ultrasound subgroups—may have limited the statistical power to detect subtle associations. Third, plasma calmodulin is not a disease-specific biomarker and may be elevated in other conditions associated with cellular injury or systemic stress. Fourth, the lack of a non-CKD control group precluded direct comparison with hypertensive or obese individuals without kidney disease. Given that hypertension alone may influence calmodulin levels, it remains unclear whether the observed associations reflect CKD-specific mechanisms or general cardiovascular stress. Fifth, the absence of renal resistive index and renin–aldosterone measurements limited our ability to assess potential associations between calmodulin and intrarenal vascular resistance. Sixth, as this was a single-center study involving mostly early-stage CKD patients with preserved renal function, the generalizability of the findings to broader or more advanced pediatric CKD populations may be limited. Finally, the cost-effectiveness and feasibility of measuring plasma calmodulin in clinical practice remain to be determined. Further studies are needed to evaluate whether the use of calmodulin as a biomarker is practical and economically justified.

## 5. Conclusions

In this study, we demonstrated that plasma calmodulin levels are independently associated with BP profiles in children with CKD, even after adjusting for conventional cardiovascular risk factors. Elevated calmodulin levels also correlated with markers of subclinical cardiovascular remodeling, including increased LV mass and carotid IMT. These findings suggest that plasma calmodulin may serve as a sensitive, though non-specific, biomarker of early CVD in pediatric CKD. Unlike traditional risk markers that may be influenced by developmental and hormonal variability in children, calmodulin retained predictive stability in our cohort. Thus, plasma calmodulin offers promise for improving early cardiovascular risk stratification in this high-risk population. Further longitudinal studies and mechanistic investigations are warranted to validate calmodulin’s clinical utility and to clarify the pathways underlying its release into circulation during CKD-related cardiovascular remodeling.

## Figures and Tables

**Table 1:** Baseline clinical and biochemical characteristics of the study participants.

Total Participants		n = 81
Baseline Characteristics	Median [IQR]	Unit
Age	9.48 [6.70, 12.86]	Years
Male	47 (58.0%)	n (%)
CAKUT	60 (74.1%)	n (%)
eGFR	104.4 [90.31, 117.2]	mL/min/1.73 m^2^
Height	138.7 [117.9, 155.9]	cm
Weight	32.7 [21.85, 44.55]	kg
BMI	17.35 [15.34, 21.15]	kg/m^2^
Underweight	9 (11.1%)	n (%)
Overweight/obesity	26 (32.1%)	n (%)
Office SBP	110.0 [101.0, 118.8]	mmHg
Office DBP	71.5 [67.0, 77.0]	mmHg
Elevated office BP/office HTN	50 (61.7%)	n (%)
LDL-C	93.0 [75.25, 118.5]	mg/dL
Triglycerides	67.0 [45.0, 97.25]	mg/dL
Fasting Plasma Glucose	85.5 [82.0, 89.75]	mg/dL
Uric Acid	4.8 [4.2, 6.1]	mg/dL
BUN	13.0 [10.0, 15.0]	mg/dL
Creatinine	0.54 [0.45, 0.67]	mg/dL
UPCR	54.9 [38.75, 93.35]	mg/g
Hemoglobin	13.1 [12.65, 14.3]	g/dL
WBC	6.6 [5.65, 8.1]	×10^3^/μL
Platelets	297.0 [245.5, 357.0]	×10^3^/μL
Sodium	140.0 [139.0, 141.0]	mEq/L
Potassium	4.4 [4.1, 4.5]	mEq/L
Calcium	10.1 [9.8, 10.4]	mg/dL
Inorganic Phosphate	4.8 [4.4, 5.1]	mg/dL
Calmodulin	137.9 [83.35, 201]	×10^2^ pg/mL

Data are presented as median [IQR] or n (%).

**Table 2:** Correlation between plasma calmodulin levels and baseline clinical and biochemical parameters in children with CKD.

Baseline Characteristics		n = 81
Parameter	r	*p* Value
Age	0.1594	0.1552
eGFR	−0.09152	0.4194
Height	0.1016	0.3701
Weight	0.2717	0.0141 (*)
BMI	0.3265	0.0031 (**)
Office SBP	−0.1003	0.4156
Office DBP	0.07021	0.5694
LDL-C	0.1113	0.3255
Triglycerides	−0.0147	0.897
Fasting Plasma Glucose	0.04954	0.6625
Uric acid	0.1273	0.2576
BUN	−0.06777	0.5478
Creatinine	0.00657	0.9536
UPCR	−0.04278	0.7045
Hemoglobin	0.2011	0.0718
WBC	0.01874	0.0939
Platelets	0.05743	0.6106
Sodium	0.01947	0.863
Potassium	0.03792	0.7368
Calcium	0.3268	0.0029 (**)
Inorganic Phosphate	−0.136	0.226

*p* < 0.05 (*), and *p* < 0.01 (**) by Pearson’s correlation analysis.

**Table 3:** Correlation between plasma calmodulin levels and cardiovascular parameters in children with CKD.

Echocardiography		n = 81
Parameter	r	*p* Value
LVPWd	0.1254	0.2678
LVIDd	0.1164	0.3038
IVSd	0.2492	0.0258 (*)
LV mass	0.2472	0.0271 (*)
FS	0.03405	0.7643
EF	0.03019	0.7904
**Carotid Artery Ultrasound**		**n = 44**
**Parameter**	**r**	***p* Value**
Left catotid IMT	0.2748	0.071
Left catotid beta	0.1433	0.3533
Left catotid AI	0.001298	0.9933
Left catotid PWV	0.1766	0.2514
Left catotid artery diameter maximum	0.08736	0.5728
Left catotid artery diameter mininum	0.06935	0.6547
Right catotid IMT	0.3836	0.0104 (**)
Right catotid beta	0.1562	0.3111
Right catotid AI	−0.01228	0.9369
Right catotid PWV	0.1721	0.2623
Right catotid artery diameter maximum	0.1086	0.4831
Right catotid artery diameter mininum	0.09321	0.5473
**ABPM**		**n = 38**
**Parameter**	**r**	***p* Value**
Overall SBP	0.4934	0.0016 (**)
Overall DBP	0.294	0.0732
Overall Mean BP	0.008937	0.9575
Overall SBP load	0.4746	0.0026 (**)
Overall DBP load	0.233	0.1592
Awake SBP	0.5094	0.0011 (**)
Awake DBP	0.3118	0.0567
Awake Mean BP	−0.01725	0.9181
Awake SBP Load	0.4955	0.0016 (**)
Awake DBP load	0.2329	0.1549
Asleep SBP	0.3798	0.0187 (*)
Asleep DBP	0.2017	0.2247
Asleep Mean BP	−0.0304	0.8562
Asleep SBP load	0.3681	0.023 (*)
Asleep DBP load	0.1892	0.2552
SBP Asleep Dipping percentage	0.138	0.4088
DBP Asleep Dipping percentage	0.06934	0.06791
AASI	0.1226	0.4634

*p* < 0.05 (*), and *p* < 0.01 (**) by Pearson’s correlation analysis.

**Table 4:** Clinical predictors of abnormal BP profile in children with CKD.

	Univariate			Multivariate		
Variable	OR	95% CI	*p* Value	Adjusted OR	95% CI	*p* Value
Age (years)	0.9588	0.8500 to 1.079	0.4855			
Male (vs. Female)	1.53	0.6170 to 3.818	0.3583			
CAKUT (vs. non-CAKUT)	1.329	0.4256 to 4.029	0.6154			
eGFR (mL/min/1.73 m^2^)	0.993	0.9730 to 1.012	0.471			
BMI (kg/m^2^)	1.158	1.021 to 1.342	0.0339 (*)	0.9376	0.7654 to 1.141	0.5188
Office SBP (mmHg)	1.1	1.044 to 1.174	0.0013 (**)	1.14	1.063 to 1.245	0.001 (**)
Office DBP (mmHg)	1.078	1.013 to 1.168	0.0361 (*)			
Fasting Plasma Glucose (mg/dL)	1.014	0.9581 to 1.077	0.6374			
LDL-C (mg/dL)	1.005	0.9914 to 1.020	0.5066			
Triglycerides (mg/dL)	0.9998	0.9931 to 1.007	0.9502			
Uric Acid (mg/dL)	1.339	1.013 to 1.916	0.0705	1.039	0.7154 to 1.611	0.8505
UPCR (mg/g)	1	0.9999 to 1.001	0.5979			
Hemoglobin (g/dL)	0.9926	0.6955 to 1.402	0.9661			
Sodium (mEq/L)	0.9575	0.7064 to 1.282	0.7721			
Potassium (mEq/L)	2.227	0.5677 to 9.800	0.2651			
Calcium (mg/dL)	1.143	0.4238 to 3.040	0.7856			
Inorganic Phosphorus (mg/dL)	1.618	0.7031 to 3.906	0.2652			
Calmodulin (×10^2^ pg/mL)	1.008	1.002 to 1.016	0.0167 (*)	1.012	1.003 to 1.024	0.029 (*)

*p* < 0.05 (*), and *p* < 0.01 (**) by univariate and multivariate logistic regression analysis.

**Table 5:** Predictive performance of plasma calmodulin levels for identifying abnormal BP profiles in children with CKD.

Calmodulin (×10^2^ pg/mL)	Abnormal BP Profile	Normal BP Profile	Total	
≥131.04	37	10	47	PPV: 78.72%
<131.04	13	21	34	NPV: 61.76%
Total	50	31		
	Sensitivity: 74%	Specificity: 67.74%		

## Data Availability

The datasets generated and analyzed during this study are not publicly accessible due to concerns regarding patient confidentiality and ethical restrictions. However, they can be obtained from the corresponding author upon reasonable request.

## Supplementary Materials

The following supporting information can be downloaded at https://www.mdpi.com/article/10.3390/children12050599/s1: Table S1: UPCR in patients with nephrotic syndrome and isolated proteinuria; Table S2: Comparison of plasma calmodulin concentrations by gender and underlying kidney disease etiology; Table S3: Variance inflation factor (VIF) analysis for variables included in the multivariate regression model; Table S4: Diagnostic performance metrics of plasma calmodulin across various cutoff values for predicting abnormal BP profiles in children with CKD.

## Abbreviations

The following abbreviations are used in this manuscript:

ABPM	ambulatory blood pressure monitoring
BMI	body mass index
BP	blood pressure
BUN	blood urea nitrogen
CAKUT	congenital anomalies of the kidney and urinary tract
CKD	chronic kidney disease
CVD	cardiovascular disease
Calmodulin	calcium-binding messenger protein
DBP	diastolic blood pressure
eGFR	estimated glomerular filtration rate
EF	ejection fraction
FS	fractional shortening
IVSd	interventricular septal thickness at end-diastole
LDL-C	low-density lipoprotein cholesterol
LV mass	left ventricular mass
LVIDd	left ventricular internal diameter at end-diastole
LVPWd	left ventricular posterior wall thickness at end-diastole
AI	augmentation index
IMT	intima–media thickness
PWV	pulse wave velocity
beta	β-stiffness index
NPV	negative predictive value
PPV	positive predictive value
Pi	inorganic phosphate
SBP	systolic blood pressure
UPCR	urinary protein-to-creatinine ratio
WBC	white blood cell count
eGFR	estimated glomerular filtration rate

## References

[1] Taşdemir M., Eroğlu A.G., Canpolat N., Konukoğlu D., Ağbaş A., Sevim M.D., Çalışkan S., Sever L. (2016). Cardiovascular alterations do exist in children with stage-2 chronic kidney disease. Clin. Exp. Nephrol..

[2] Schaefer F., Doyon A., Azukaitis K., Bayazit A., Canpolat N., Duzova A., Niemirska A., Sözeri B., Thurn D., Anarat A. (2017). Cardiovascular Phenotypes in Children with CKD: The 4C Study. Clin. J. Am. Soc. Nephrol..

[3] Daniels S.R., Kimball T.R., Morrison J.A., Khoury P., Meyer R.A. (1995). Indexing left ventricular mass to account for differences in body size in children and adolescents without cardiovascular disease. Am. J. Cardiol..

[4] Mitsnefes M.M., Kimball T.R., Kartal J., Witt S.A., Glascock B.J., Khoury P.R., Daniels S.R. (2006). Progression of left ventricular hypertrophy in children with early chronic kidney disease: 2-year follow-up study. J. Pediatr..

[5] Samuels J., Ng D., Flynn J.T., Mitsnefes M., Poffenbarger T., Warady B.A., Furth S. (2012). Ambulatory blood pressure patterns in children with chronic kidney disease. Hypertension.

[6] Tain Y.-L., Hsu C.-N. (2022). Cardiovascular Risks of Hypertension: Lessons from Children with Chronic Kidney Disease. Children.

[7] Chien S.-J., Lin I.C., Hsu C.-N., Lo M.-H., Tain Y.-L. (2015). Homocysteine and Arginine-to-Asymmetric Dimethylarginine Ratio Associated with Blood Pressure Abnormalities in Children With Early Chronic Kidney Disease. Circ. J..

[8] Wang S., Vicente F.B., Miller A., Brooks E.R., Price H.E., Smith F.A. (2007). Measurement of arginine derivatives in pediatric patients with chronic kidney disease using high-performance liquid chromatography-tandem mass spectrometry. Clin. Chem. Lab. Med..

[9] Mitsnefes M.M., Betoko A., Schneider M.F., Salusky I.B., Wolf M.S., Jüppner H., Warady B.A., Furth S.L., Portale A.A. (2018). FGF23 and Left Ventricular Hypertrophy in Children with CKD. Clin. J. Am. Soc. Nephrol..

[10] Grund A., Sinha M.D., Haffner D., Leifheit-Nestler M. (2021). Fibroblast Growth Factor 23 and Left Ventricular Hypertrophy in Chronic Kidney Disease-A Pediatric Perspective. Front. Pediatr..

[11] Sinha M.D., Turner C., Booth C.J., Waller S., Rasmussen P., Goldsmith D.J.A., Simpson J.M. (2015). Relationship of FGF23 to indexed left ventricular mass in children with non-dialysis stages of chronic kidney disease. Pediatr. Nephrol..

[12] Chen W.L., Tain Y.L., Chen H.E., Hsu C.N. (2021). Cardiovascular Disease Risk in Children with Chronic Kidney Disease: Impact of Apolipoprotein C-II and Apolipoprotein C-III. Front. Pediatr..

[13] Holle J., Querfeld U., Kirchner M., Anninos A., Okun J., Thurn-Valsassina D., Bayazit A., Niemirska A., Canpolat N., Bulut I.K. (2019). Indoxyl sulfate associates with cardiovascular phenotype in children with chronic kidney disease. Pediatr. Nephrol..

[14] Means A.R. (1981). Calmodulin: Properties, Intracellular Localization, and Multiple Roles in Cell Regulation. Recent Prog. Horm. Res..

[15] Sperelakis N. (1990). Properties of calcium channels in cardiac muscle and vascular smooth muscle. Mol. Cell. Biochem..

[16] Francis A., Shroff R., Earley A., Foster B.J. (2025). KDIGO 2024 Guidelines—Key Points for Pediatricians. JAMA Pediatr..

[17] Höcht C. (2013). Blood Pressure Variability: Prognostic Value and Therapeutic Implications. Int. Sch. Res. Not..

[18] Schutte A.E., Kollias A., Stergiou G.S. (2022). Blood pressure and its variability: Classic and novel measurement techniques. Nat. Rev. Cardiol..

[19] Levey A.S., Coresh J., Bolton K., Culleton B., Harvey K.S., Ikizler T.A., Johnson C.A., Kausz A., Kimmel P.L., Kusek J. (2002). K/DOQI clinical practice guidelines for chronic kidney disease: Evaluation, classification, and stratification. Am. J. Kidney Dis..

[20] Schwartz G.J., Muñoz A., Schneider M.F., Mak R.H., Kaskel F., Warady B.A., Furth S.L. (2009). New equations to estimate GFR in children with CKD. J. Am. Soc. Nephrol..

[21] Health Promotion Administration, Ministry of Health and Welfare Recommended BMI Reference Values for Growth in Children and Adolescents. https://www.hpa.gov.tw/542/9547/n.

[22] Flynn J.T., Kaelber D.C., Baker-Smith C.M., Blowey D., Carroll A.E., Daniels S.R., de Ferranti S.D., Dionne J.M., Falkner B., Flinn S.K. (2017). Clinical Practice Guideline for Screening and Management of High Blood Pressure in Children and Adolescents. Pediatrics.

[23] Mac Neil S., Walker S.W., Seid J., Tomlinson S. (1984). Calmodulin in human serum and the specific release of calmodulin from calmodulin-rich platelets. Biosci. Rep..

[24] Houston D.S., Carson C.W., Esmon C.T. (1997). Endothelial cells and extracellular calmodulin inhibit monocyte tumor necrosis factor release and augment neutrophil elastase release. J. Biol. Chem..

[25] Ono K., Niwa M., Suzuki H., Kobayashi N.B., Yoshida T., Sawada M. (2022). Calmodulin as a Key Regulator of Exosomal Signal Peptides. Cells.

[26] Esteras N., Alquézar C., de la Encarnación A., Villarejo A., Bermejo-Pareja F., Martín-Requero A. (2013). Calmodulin levels in blood cells as a potential biomarker of Alzheimer’s disease. Alzheimers Res. Ther..

[27] Prasad A.M., Morgan D.A., Nuno D.W., Ketsawatsomkron P., Bair T.B., Venema A.N., Dibbern M.E., Kutschke W.J., Weiss R.M., Lamping K.G. (2015). Calcium/calmodulin-dependent kinase II inhibition in smooth muscle reduces angiotensin II-induced hypertension by controlling aortic remodeling and baroreceptor function. J. Am. Heart Assoc..

[28] Muthalif M.M., Karzoun N.A., Benter I.F., Gaber L., Ljuca F., Uddin M.R., Khandekar Z., Estes A., Malik K.U. (2002). Functional significance of activation of calcium/calmodulin-dependent protein kinase II in angiotensin II--induced vascular hyperplasia and hypertension. Hypertension.

[29] Usui T., Okada M., Hara Y., Yamawaki H. (2011). Exploring calmodulin-related proteins, which mediate development of hypertension, in vascular tissues of spontaneous hypertensive rats. Biochem. Biophys. Res. Commun..

[30] Huang S.L., Wen Y.I., Kupranycz D.B., Pang S.C., Schlager G., Hamet P., Tremblay J. (1988). Abnormality of calmodulin activity in hypertension. Evidence of the presence of an activator. J. Clin. Investig..

[31] Zuidscherwoude M., Grigore T., van de Langenberg B., Witte G., van der Wijst J., Hoenderop J.G. (2024). Calmodulin regulates TRPV5 intracellular trafficking and plasma membrane abundance. J. Physiol..

[32] Basu U., Case A.J., Liu J., Tian J., Li Y.L., Zimmerman M.C. (2019). Redox-sensitive calcium/calmodulin-dependent protein kinase IIα in angiotensin II intra-neuronal signaling and hypertension. Redox Biol..

[33] Wang Y., Tandan S., Cheng J., Yang C., Nguyen L., Sugianto J., Johnstone J.L., Sun Y., Hill J.A. (2008). Ca^2+^/calmodulin-dependent protein kinase II-dependent remodeling of Ca^2+^ current in pressure overload heart failure. J. Biol. Chem..

[34] Schulman H., Anderson M.E. (2010). Ca/Calmodulin-dependent Protein Kinase II in Heart Failure. Drug Discov. Today Dis. Mech..

[35] Swaminathan P.D., Purohit A., Hund T.J., Anderson M.E. (2012). Calmodulin-dependent protein kinase II: Linking heart failure and arrhythmias. Circ. Res..

