# Supplementary material for: Plasma Calmodulin as a Biomarker of Subclinical Cardiovascular Disease in Pediatric Chronic Kidney Disease
Source: Children (Basel). 2025 May 4;12(5):599. doi: 10.3390/children12050599 (PMC12109911; doi:10.3390/children12050599)
Supplement: Supplementary file 1 [file children-12-00599-s001.zip › children-3596949-supplementary.pdf]

Table S1. Urine protein-to-creatinine ratio (UPCR) in patients with nephrotic syndrome and isolated proteinuria

Group	Number of patients (n)	Median UPCR (mg/mg)	IQR (mg/mg)
Nephrotic syndrome	8	58.45	45.88–1320.28
Isolated proteinuria	4	350.45	317.38–541.60

Table S2. Comparison of plasma calmodulin concentrations by gender and underlying kidney disease etiology. Data are presented as median (interquartile range). $P < 0.05$ (*), $P < 0.01$ (**), and $P < 0.001$ (***) by the Mann–Whitney U test.

	**Female (n=34)**	**Male (n=47)**	**P value**
Calmodulin (×10^2 pg/mL)	140.78 (87.94, 203.33)	138.11 (82.09, 200.80)	0.981
	**CAKUT (n=60)**	**Non-CAKUT (n=21)**	**P value**
Calmodulin (×10^2 pg/mL)	144.14 (84.92, 201.55)	112.36 (82.99, 184.41)	0.5525

Table S3. Variance inflation factor (VIF) analysis for variables included in the multivariate regression model.

Variable	VIF
BMI (kg/m^2)	1.256
Uric Acid (mg/dL)	1.089
Office SBP (mmHg)	1.339
Office DBP (mmHg)	1.259
Calmodulin (×10^2 pg/mL)	1.024

Table S4. Diagnostic performance metrics of plasma calmodulin across various cutoff values for predicting abnormal blood pressure profiles in children with chronic kidney disease. Sensitivity, specificity, and Youden index (sensitivity + specificity – 1) are listed for each threshold.

Calmodulin (×10^2 pg/mL)	Sensitivity	Specificity	Youden Index
131.03889	0.74	0.677419355	0.417419355
113.79834	0.76	0.64516129	0.40516129
132.99287	0.72	0.677419355	0.397419355
127.69737	0.74	0.64516129	0.38516129
133.15199	0.7	0.677419355	0.377419355
112.75199	0.76	0.612903226	0.372903226
133.40699	0.68	0.677419355	0.357419355
112.41284	0.76	0.580645161	0.340645161
135.80093	0.66	0.677419355	0.337419355
110.54777	0.78	0.548387097	0.328387097
137.61432	0.64	0.677419355	0.317419355
156.38878	0.54	0.774193548	0.314193548
112.35708	0.76	0.548387097	0.308387097
148.54702	0.56	0.741935484	0.301935484
138.11021	0.62	0.677419355	0.297419355
102.42687	0.78	0.516129032	0.296129032
162.26568	0.52	0.774193548	0.294193548
144.67799	0.58	0.709677419	0.289677419
149.18452	0.54	0.741935484	0.281935484
143.95226	0.6	0.677419355	0.277419355
163.76933	0.5	0.774193548	0.274193548
92.74027	0.82	0.451612903	0.271612903
145.47121	0.56	0.709677419	0.269677419
82.25926	0.88	0.387096774	0.267096774
98.96516	0.78	0.483870968	0.263870968
85.41565	0.84	0.419354839	0.259354839
144.31861	0.58	0.677419355	0.257419355
166.00126	0.48	0.774193548	0.254193548
94.60058	0.8	0.451612903	0.251612903
69.09752	0.96	0.290322581	0.250322581
83.71973	0.86	0.387096774	0.247096774
76.74718	0.92	0.322580645	0.242580645

88.77655	0.82	0.419354839	0.239354839
63.77125	0.98	0.258064516	0.238064516
82.08671	0.88	0.35483871	0.23483871
167.33559	0.46	0.774193548	0.234193548
98.24045	0.78	0.451612903	0.231612903
70.35603	0.94	0.290322581	0.230322581
84.7569	0.84	0.387096774	0.227096774
78.12435	0.9	0.322580645	0.222580645
66.04959	0.96	0.258064516	0.218064516
180.35929	0.44	0.774193548	0.214193548
72.00792	0.92	0.290322581	0.210322581
62.6246	0.98	0.225806452	0.205806452
79.0857	0.88	0.322580645	0.202580645
181.71657	0.42	0.774193548	0.194193548
54.82058	1	0.193548387	0.193548387
190.54705	0.38	0.806451613	0.186451613
182.78009	0.4	0.774193548	0.174193548
60.65651	0.98	0.193548387	0.173548387
191.29692	0.36	0.806451613	0.166451613
52.70153	1	0.161290323	0.161290323
185.67315	0.38	0.774193548	0.154193548
269.47261	0.18	0.967741935	0.147741935
192.04968	0.34	0.806451613	0.146451613
247.55451	0.2	0.935483871	0.135483871
51.94146	1	0.129032258	0.129032258
291.77032	0.16	0.967741935	0.127741935
192.94745	0.32	0.806451613	0.126451613
242.77598	0.22	0.903225806	0.123225806
368.42706	0.12	1	0.12
265.08916	0.18	0.935483871	0.115483871
319.52452	0.14	0.967741935	0.107741935
200.80077	0.3	0.806451613	0.106451613
243.28462	0.2	0.903225806	0.103225806
386.43091	0.1	1	0.1
51.84133	1	0.096774194	0.096774194
223.85651	0.22	0.870967742	0.090967742
344.25833	0.12	0.967741935	0.087741935

201.4857	0.28	0.806451613	0.086451613
466.23418	0.08	1	0.08
220.32017	0.24	0.838709677	0.078709677
201.80326	0.26	0.806451613	0.066451613
41.25832	1	0.064516129	0.064516129
783.2512	0.06	1	0.06
221.48229	0.22	0.838709677	0.058709677
207.89419	0.24	0.806451613	0.046451613
915.49896	0.04	1	0.04
41.04905	1	0.032258065	0.032258065
959.11671	0.02	1	0.02
26.90131	1	0	0
